# Adequate Prenatal Care Reduces the Risk of Adverse Pregnancy Outcomes in Women with History of Infertility: A Nationwide Population-Based Study

**DOI:** 10.1371/journal.pone.0084237

**Published:** 2013-12-17

**Authors:** Raushan Alibekova, Jian-Pei Huang, Yi-Hua Chen

**Affiliations:** 1 School of Public Health, College of Public Health and Nutrition, Taipei Medical University, Taipei, Taiwan; 2 Department of Obstetrics and Gynecology, Mackay Memorial Hospital, Taipei, Taiwan; University of Missouri, United States of America

## Abstract

**Objectives:**

To investigate the effects of various measures of prenatal care on adverse pregnancy outcomes in women with a history of infertility.

**Study Design:**

A retrospective cohort study.

**Methods:**

Data were derived by linking 2 large nationwide population-based datasets, the National Health Insurance Research Database and Taiwan Birth Certificate Registry. The study sample included 15,056 women with an infertility diagnosis and 60,224 randomly selected women without infertility matched to the study sample by maternal age. A conditional logistic regression analysis was performed for the analysis.

**Results:**

Women diagnosed with infertility respectively had 1.39 (95% CI, 1.06~1.83), 1.15 (95% CI, 1.08~1.24), 1.13 (95% CI, 1.08~1.18), and 1.08 (95% CI, 1.05~1.12) higher odds of having very low birth weight (VLBW) babies, preterm births, labor complications, and cesarean sections (CSs) compared to women without infertility. Inadequate numbers of total and major prenatal visits and late initiation of prenatal care increased the risks of adverse pregnancy outcomes in women with infertility, especially the risk of a VLBW baby. However, no significant associations were found for the risks of adverse birth outcomes in infertile women with adequate prenatal care compared to fertile women with adequate care.

**Conclusions:**

Study findings suggest that adequate prenatal care can reduce the risk of adverse pregnancy outcomes in women with infertility.

## Introduction

Prenatal care is recognized as a key population-wide public health intervention to prevent adverse pregnancy outcomes. Under-attending antenatal care appears to be a significant contributor to low birth weight (LBW) babies, preterm birth, obstetric complications, and neonatal mortality [1-4]. Extensive use of prenatal care is often linked to high-risk pregnancies [3]; however the impacts of prenatal care on adverse pregnancy outcomes in high-risk pregnancies have not been widely explored.

Appropriate prenatal screening and counseling are of crucial importance for pregnant women with a history of infertility; these women are mostly older than average pregnant women, have a greater number of chronic conditions, and are at risk for spontaneous pregnancy loss and chromosomal abnormalities [5,6]. Studies showed that even after adjusting for age and parity, subfertile women are at higher risk of preeclampsia, placenta previa, and placental abruption, and are more likely to undergo induction of labor, have a cesarean section (CS), and deliver LBW and preterm infants [7,8].

Previous studies showed that women with singleton births who used various treatment modalities for infertility, including assisted reproductive technologies (ARTs), have worse pregnancy outcomes despite the adequate use of prenatal care, compared to the general population [9,10]. However, impacts of prenatal care on adverse birth and obstetric outcomes in infertile women have seldom been investigated. In a related study by Wehby et al. [11], the effect of prenatal care use on birth outcomes was evaluated after accounting for women’s health and fertility characteristics. The findings of that study showed that delays in receiving prenatal care increased the preterm birth risk, while more-frequent use of prenatal care significantly improved the birth weight among pregnancies at high risk for an LBW baby, including women with low fertility. Those findings have important health policy implications for improving birth outcomes in high-risk pregnancies. However, that was a clinic-based study, and the ability to generalize its findings to the population as a whole is limited due to a potential selection bias. In addition, that study only examined the effect of utilization measures of prenatal care on birth outcomes. Investigation of the risk-appropriate content, and frequency and timing of prenatal care is needed to improve adverse pregnancy outcomes [12]. Most previous studies on the effectiveness of prenatal care lacked data on the quality or content of prenatal care.

To investigate the association between prenatal care and adverse pregnancy outcomes in women with a history of infertility, we undertook a retrospective cohort analysis, linking 2 large nationwide population-based datasets. The objectives of our study were to: (1) compare prenatal care visits between women with and without an infertility diagnosis, (2) determine associations of infertility with adverse pregnancy outcomes, labor complications, and a CS, and (3) examine the relationship between inadequate prenatal care and adverse pregnancy outcomes in women diagnosed with infertility compared to women without infertility. This is the largest population-based study in Taiwan to examine the effect of prenatal care on adverse outcomes among high-risk pregnancies in infertile women.

## Methods

### Data sources

Data from 2 nationwide population-based datasets in Taiwan were analyzed. The first dataset, the National Health Insurance (NHI) Research Database (NHIRD), is derived from the Taiwanese NHI program, launched in 1995 to finance health care for all citizens. We used the NHIRD which includes all inpatient and ambulatory care medical claims for 2005. The system provides care for almost 99% of the Taiwanese population of over 23 million people, and offers a unique opportunity for research purposes.

The second dataset, the Taiwan National Birth Certificate Registry (NBCR), is published by the Ministry of the Interior. This dataset contains both infant and parental information, including pregnancy outcomes and sociodemographic characteristics. The requirement for compulsory registration of all births and deaths in Taiwan ensures the accuracy and comprehensiveness of the birth certificate data.

The 2 datasets are linked, and all personal identifiers are removed before data are released to researchers. Since this study employed de-identified secondary data, it was exempt from full review by the Taipei Medical University Institutional Review Board.

### Study design and sample

A retrospective cohort design was utilized. In total, 213,206 women with singleton live births, who were pregnant and attended prenatal visits in the NHI program from 1 January to 31 December 2005, were identified. Among these women, we identified 15,056 women who had been diagnosed with infertility (International Classification of Diseases, Ninth Revision, Clinical Modification (ICD-9-CM) code 628), and had at least 2 consensus diagnoses within 3 years prior to the current delivery to ensure the validity of diagnosis. Out of the remaining 198,150 women, a comparison cohort of 60,224 women was randomly selected (4 for every woman with infertility treatment) and matched to the study cohort by age categories (<20, 20~24, 25~29, 30~34, 35~39, and ≥40 years). We further ensured that none of the women in the comparison cohort had been diagnosed with infertility since 1995, when the NHI program was initiated.

### Variables of interest

To fulfill our first objective, we compared the prenatal care of women with and without a history of infertility. The key independent variable was whether or not a pregnant woman had been diagnosed with infertility.

Dependent variables included dichotomous outcome measures of the number of prenatal care visits, initiation of prenatal care, and utilization of major prenatal visits as a proxy measure for the content of prenatal care visits. The number, timing, and contents of prenatal care are widely recommended measures for assessing prenatal care [13].

In Taiwan, 10 prenatal visits are recommended by the Department of Health, provided free of charge, for all Taiwanese women in order to improve the accessibility to prenatal care and reduce the risk of poor pregnancy outcomes. The covered prenatal care services vary according to the trimester [14,15]. During the first trimester, 2 visits are recommended, and covered prenatal services include routine history taking and diagnostic questioning, a physical examination, and laboratory tests (regular blood tests, blood type, Rh factor, syphilis screening, rubella immunoglobulin G, an AIDS test, and regular urine tests). Prenatal care services during the second trimester include regular checkup examinations (diagnostic questioning, a physical examination, and urine protein and glucose tests), and ultrasound (US) screening. Two visits are recommended during the second trimester. Prenatal services during the third trimester include regular checkup examinations, and additional laboratory tests provided around the 32^nd^ week of pregnancy (hepatitis B antibody test, HBsAG, HBeAG, and VDRL laboratory tests). Up to 6 visits are covered by the NHI during the third trimester. For high-risk pregnant women who receive more than 10 prenatal checkups and 1 US screening, additional self-paid services are available [15].

According to an adequacy of prenatal care utilization index, late initiation (after the fourth month of pregnancy) or receiving fewer than 50% of the recommended visits is considered inadequate utilization [16]. For our high-risk pregnancy cohort of infertile women, we categorized the number of prenatal visits of <6 as inadequate. In our study, initiation of prenatal care after the first trimester (>12 weeks) of pregnancy was categorized as late, based on previous research that used a similar definition to identify timely and untimely prenatal care for women with high-risk pregnancies [17]. Contents of prenatal care include physical assessments, laboratory tests, US examinations, and other medical procedures [18]. A previous study suggested measuring the content of prenatal care by initial prenatal procedures performed during the first 2 visits [19]. In our study, we considered the first 5 prenatal visits, which are performed during the first 32 weeks of pregnancy to ensure the health of the fetus in the early developmental stage, as major visits. These major prenatal visits with important blood and urine tests for risk assessments, and US screening for gestational age and fetal anomalies, served as a proxy measure for the content of prenatal care in the present study. The number of major prenatal visits participated in was categorized as adequate (3–5) or inadequate (≤2).

To examine the relationship between infertility and adverse pregnancy outcomes, our dependent variables were dichotomous outcome measures including very LBW (VLBW; <1500 g), LBW (<2500 g), preterm birth (<37 weeks), and congenital malformations (e.g., heart defects, a cleft lip or palate, Down syndrome, spina bifida, limb defects, etc.). Other dependent variables included labor complications (e.g., premature rupture of the membrane of >12 h, prolonged labor with regular uterus contractions of >20 h, precipitous labor of <3 h, placenta or previa abruption, etc.), and a CS (ICD-9-CM code 669.7).

Several covariates were considered in our regression modeling. Sociodemographic covariates included the educational level of the mother, family monthly income, urbanization level of the place of residence, and parity. The history of comorbid medical conditions included hyperlipidemia, type 2 diabetes, thyroid dysfunction, urinary tract infections (UTIs), deficiency anemia, and depression. The pregnancy history included gestational hypertension and preeclampsia, diabetes mellitus, coronary heart disease, and a previous CS (ICD-9-CM codes for the above mentioned medical conditions are provided in the Table S1).

### Statistical analysis

Descriptive statistics were performed, with percentages for categorical data, and means and standard deviations (SDs) for continuous data. Differences between exposed and unexposed cohorts were assessed using χ^2^ or *t*-tests depending on whether the variable was categorical or continuous. A conditional logistic regression was used to assess the relationship between an infertility diagnosis and 2 groups of outcome variables including prenatal care visits and adverse pregnancy outcomes. Specifically, we first estimated crude odd ratios (ORs) from a simple conditional logistic regression that took into account the matching variable of age in the analysis. Then we adjusted for all of the risk factors investigated in our conditional logistic regression model, including maternal demographics, chronic medical conditions, and pregnancy history. We also investigated the effects of an infertility diagnosis, combined with levels of prenatal care received, on adverse pregnancy outcomes. The adequacy of prenatal care among fertile and infertile women was assessed by the number of prenatal visits, timing of prenatal visits, and major prenatal care visits. All analyses were performed using statistical software SAS, vers. 9.3 (SAS, Cary, NC, USA). Significance was set at two-tailed *p*<0.05.

## Results

Descriptive characteristics of pregnant women with and without a history of infertility are presented in Table 1. Women who had been diagnosed with infertility within 3 years prior to the current pregnancy had a higher income, were residents of more-urbanized areas, and were more highly educated, compared to women without an infertility diagnosis (*p*<0.0001). They also had a higher prevalence of chronic conditions, such as hyperlipidemia, type 2 diabetes, thyroid dysfunction, deficiency anemia, and UTIs, than comparison subjects (*p*<0.001). There was a statistically significant difference in the number of major prenatal care visits made between the 2 study cohorts (*p*<0.0001), and the mean number of total prenatal care visits was significantly higher in the infertility-diagnosed cohort compared to unaffected women (8.2 vs. 7.9, respectively, *p*<0.0001).

**Table 1:** Selected characteristics of women with and without a history of an infertility diagnosis in Taiwan in 2005 (*n*=75,280).

Variable		**Women without an infertility diagnosis (*n*=60,224)**		**Women with an infertility diagnosis (*n*=15,056)**	*p* value ^a^
		No. (%)		No. (%)	
**Sociodemographics**					
Age (years)					1.0000
<20		80 (0.1)		20 (0.1)	
20~24		3108 (5.2)		777 (5.2)	
25~39		17,976 (29.9)		4494 (29.9)	
30~34		25,960 (43.1)		6490 (43.1)	
35~39		11,692 (19.4)		2923 (19.4)	
≥40		1408 (2.3)		352 (2.3)	
Family income (NTD ^b^/month)					<0.0001
<15,000		18,107 (30.1)		4058 (27.0)	
15,000~30,000		24,702 (41.1)		6461 (42.9)	
31,001~50,000		13,593(22.6)		3398 (22.6)	
>50,000		3822 (6.4)		1139 (7.6)	
Education					<0.0001
Less than high school		4642 (7.7)		898 (6.0)	
High school		21,929 (36.4)		5487 (36.4)	
College		30,755 (51.1)		7912 (52.6)	
Graduate school		2840 (4.7)		736 (4.9)	
Urbanization					<0.0001
1 (most)		18,508 (30.7)		4750 (31.6)	
2		30,126 (50.0)		7757 (51.5)	
3		6999 (11.6)		1564 (10.4)	
4 (least)		4591 (7.6)		985 (6.5)	
Parity					<0.0001
First child		26,663 (44.3)		8463 (56.2)	
Second child		24,560 (40.8)		5467 (36.3)	
Third child or above		9001 (14.9)		1126 (7.5)	
**Medical history**					
Hypertension					0.1025
Yes		296 (0.5)		90 (0.6)	
No		59,928 (99.5)		14,966 (99.4)	
Cardiac arrhythmias					0.0503
Yes		486 (0.8)		146 (1.0)	
No		59,738 (99.2)		14,910 (99.0)	
Hyperlipidemia					0.0003
Yes		382 (0.6)		137 (0.9)	
No		59,842 (99.4)		14,919 (99.1)	
Type 2 diabetes					<0.0001
Yes		287 (0.5)		155 (1.0)	
No		59,937 (99.5)		14,901 (99.0)	
Thyroid dysfunction					<0.0001
Yes		870 (1.4)		367 (2.4)	
No		59,354 (98.6)		14,689 (97.6)	
Deficiency anemia					<0.0001
Yes		488 (0.8)		184 (1.2)	
No		59,736 (99.2)		14,872 (98.8)	
Urinary tract infection					<0.0001
Yes		4288 (7.1)		1578 (10.5)	
No		55,936 (92.9)		13,478 (89.5)	
Depression					0.0930
Yes		756 (1.3)		215 (1.4)	
No		59,468 (98.7)		14,841 (98.6)	
**Prenatal care**					
No. of major prenatal care visits					<0.0001
5		39,561 (65.7)		10,506 (69.8)	
3 or 4		19,357 (32.1)		4380 (29.1)	
≤2		1306 (2.2)		170 (1.1)	
Total no. of prenatal visits ^c^		7.9 (2.22)		8.2 (2.13)	<0.0001

^a^ Results of χ^2^ tests for categorical variables are presented.

^b^ According to the American Institute in Taiwan website, in 2005, the exchange rate was US$1.00≈New Taiwanese Dollars (NTD)30.0.

^c^ An independent *t*-test for continuous normal distributed variables was used. Values are presented as the mean (standard deviation).

Table 2 presents the results of the conditional logistic regression analysis for relationships between an infertility diagnosis of women and prenatal care visits. Having a diagnosis of infertility was independently associated with a lower likelihood of attending prenatal care <6 times (OR 0.89, 95% confidence interval (CI) 0.86~0.93), initiating the first prenatal visit after 12 weeks of gestation (OR 0.74, 95% CI 0.70~0.78), and receiving ≤2 major prenatal visits (OR 0.56, 95% CI, 0.48~0.66).

**Table 2:** Crude and adjusted odds ratios (ORs) of inadequate prenatal care in relation to an infertility diagnosis of women in Taiwan in 2005 (*n*=75,280).

Variable	**Without an infertility diagnosis** (*n*=60,224)	**With an infertility diagnosis** (*n*=15,056)
	No. (%)	No. (%)
**Number of prenatal visits**		
<6	11,764 (19.5)	2541 (16.9)
≥6	48,460(80.5)	12,515 (83.1)
Crude OR (95% CI)	1.00	0.86*** (0.83~0.90)
Adjusted OR ^a^ (95% CI)	1.00	0.89*** (0.86~0.93)
**Initiation of prenatal care**		
>12 weeks of pregnancy	8990 (14.9)	1559 (10.3)
≤12 weeks of pregnancy	51,234 (85.1)	1397 (89.7)
Crude OR (95% CI)	1.00	0.69*** (0.66~0.73)
Adjusted OR ^a^ (95% CI)	1.00	0.74*** (0.70~0.78)
**No. of major prenatal visits**		
≤2	1306 (2.2)	170 (1.1)
3~5	58,918 (97.8)	14,886 (98.9)
Crude OR (95% CI)	1.00	0.52*** (0.44-0.61)
Adjusted OR ^a^ (95% CI)	1.00	0.56*** (0.48-0.66)

CI, confidence interval.

^a^ Adjusted for maternal demographics (education, family income, urbanization level, and parity), comorbid chronic conditions (hyperlipidemia, thyroid dysfunction, urinary tract infections, deficiency anemia, and depression), and pregnancy history (hypertension, preeclampsia, diabetes mellitus, heart disease, and a previous cesarean section).

^***^ *p*<0.001.

The conditional logistic regression analysis of adverse birth and obstetrical outcomes in association with an infertility diagnosis of the mother (Table 3) revealed that the study cohort respectively had 1.39 (95% CI, 1.06~1.83), 1.15 (95% CI, 1.08~1.24), 1.13 (95% CI, 1.08~1.18), and 1.08 (95% CI, 1.05~1.12) higher odds of having VLBW babies, a preterm birth, labor complications, and a CS than the comparison cohort, after adjusting for potential confounders, including the total number of prenatal care visits. Our findings showed no significant associations between an infertility diagnosis and congenital malformations.

**Table 3:** Crude and adjusted odds ratios (ORs) of adverse pregnancy outcomes, labor complications, and a cesarean section according to an infertility diagnosis of mothers in Taiwan in 2005 (*n*=75,280).

Variable	**Without an infertility diagnosis** (*n*=60,224)	**With an infertility diagnosis** (*n*=15,056)
	No. (%)	No. (%)
**Very low birth weight**		
Yes	227 (0.4)	71 (0.5)
No	59,997 (99.6)	14,985 (99.5)
Crude OR (95% CI)	1.00	1.25 (0.96~1.63)
Adjusted OR ^a^ (95% CI)	1.00	1.39* (1.06~1.83)
**Low birth weight**		
Yes	3048 (5.1)	812 (5.4)
No	57,176 (94.9)	14,244 (94.6)
Crude OR (95% CI)	1.00	1.07 (0.99~1.15)
Adjusted OR ^a^ (95% CI)	1.00	1.058 (0.98~1.15)
**Preterm birth**		
Yes	3975 (6.6)	1080 (7.2)
No	56,249 (93.4)	13,976 (92.8)
Crude OR (95% CI)	1.00	1.09* (1.02~1.16)
Adjusted OR ^a^ (95% CI)	1.00	1.15*** (1.08~1.24)
**Congenital malformations**		
Yes	72 (0.1)	21 (0.1)
No	60,152 (99.9)	15,035 (99.9)
Crude OR (95% CI)	1.00	1.17 (0.72~1.90)
Adjusted OR ^a^ (95% CI)	1.00	1.10 (0.68~1.81)
**Labor complications**		
Yes	9870 (16.4)	3021 (20.1)
No	50,354 (83.6)	12,035 (79.9)
Crude OR (95% CI)	1.00	1.22*** (1.18~1.28)
Adjusted OR ^a^ (95% CI)	1.00	1.13*** (1.08~1.18)
**Cesarean section**		
Yes	21,813 (36.2)	6237 (41.4)
No	38,411 (63.8)	8819 (58.6)
Crude OR (95% CI)	1.00	1.14*** (1.11~1.18)
Adjusted OR ^a^ (95% CI)	1.00	1.08*** (1.05~1.12)

CI, confidence interval.

^a^ Adjusted for maternal demographics (education, family income, urbanization level, and parity), comorbid chronic conditions (hyperlipidemia, thyroid dysfunction, urinary tract infections, deficiency anemia, and depression), pregnancy history (hypertension, preeclampsia, diabetes mellitus, heart disease, and a previous cesarean section), and total number of prenatal care visits.

* *p*<0.05, *** *p*<0.001.

Further examination of the effects of an infertility diagnosis, combined with adequate prenatal care, on adverse pregnancy outcomes was conducted by a conditional logistic regression analysis. A cohort of fertile women who received adequate prenatal care (≥6 prenatal visits) was the reference group in our analysis. Women with an infertility diagnosis who received <6 prenatal care visits respectively had 18.15- (95% CI 12.33~26.70), 2.05- (95% CI 1.79~2.35), and 2.24-times (95% CI 1.99~2.51) higher odds of having VLBW, LBW, and preterm babies, than fertile women with an adequate number of prenatal visits. Fertile women with an inadequate number of prenatal visits were respectively 13.56- (95% CI 9.96~18.54), 1.94- (95% CI 1.80~2.10), and 1.91-times (95% CI 1.79~2.04) more likely to have VLBW, LBW, and preterm infants than the comparison group. However, no significant associations were found for risks of adverse birth outcomes in infertile women who received an adequate number of prenatal visits, compared to fertile women with adequate prenatal care. Similar trends for the risks of adverse pregnancy outcomes were also observed when the effects of an infertility diagnosis were combined with other indicators of inadequate prenatal care (i.e., initiation of prenatal care after 12 weeks of pregnancy and ≤2 major prenatal visits) (data not shown in table).

Finally, a subsample of women with an infertility diagnosis was further analyzed to estimate associations of the level of prenatal care with adverse pregnancy outcomes and birth complications (Table 4). A logistic regression analysis revealed that infertile women who had <6 prenatal care visits were respectively 15.09- (95% CI, 8.82~25.82), 2.12- (95% CI 1.81~2.49), and 2.24-times (95% CI, 1.95~2.58) more likely to have VLBW, LBW, or preterm babies compared to infertile women who had ≥6 prenatal care visits, after adjusting for potential confounders. Late initiation of prenatal care, after 12 weeks of gestation, increased the risk of having a VLBW and LBW baby (OR 2.09, 95% CI 1.16~3.79 and 1.58, CI 1.29~1.94, respectively), compared to infertile women with timely prenatal care. Infertile women who received ≤2 major prenatal visits respectively had 4.12- (95% CI 1.26~13.43) and 1.62-times (95% CI 1.16~2.27) higher risk of having a VLBW infant and labor complications compared to women diagnosed with infertility who received adequate prenatal care. On the other hand, infertile women with <6 prenatal visits had a 20% (95% CI 0.71~0.90) lower risk of having labor complications compared to infertile women with an adequate number of prenatal visits.

**Table 4:** Crude and adjusted odds ratios (ORs) for adverse pregnancy outcomes and birth complications by prenatal care among women diagnosed with infertility in Taiwan in 2005~2006 (*n*=15,056).

	**Women with an infertility diagnosis (*n*=15,056)**		
Variable	**Model 1** <6 visits vs. ≥6 prenatal visits (ref.)	**Model 2** >12 weeks vs. ≤12 weeks of pregnancy (ref.)	**Model 3** ≤2 major visits vs. 3~5 major prenatal visits (ref.)
**Very LBW**			
Crude OR (95% CI)	13.74*** (8.11~23.28)	2.14* (1.19~3.84)	3.92* (1.22~12.57)
Adjusted OR ^a^ (95% CI)	15.09*** (8.82~25.82)	2.09* (1.16~3.79)	4.12* (1.26~13.43)
**LBW**			
Crude OR (95% CI)	2.14*** (1.82~2.50)	1.53*** (1.25~1.87)	1.34 (0.74~2.42)
Adjusted OR ^a^ (95% CI)	2.12*** (1.81~2.49)	1.58*** (1.29~1.94)	1.26 (0.69~2.30)
**Preterm birth**			
Crude OR (95% CI)	2.22*** (1.93~2.55)	1.078 (0.88~1.31)	1.64* (1.01~2.65)
Adjusted OR ^a^ (95% CI)	2.24*** (1.95~2.58)	1.068 (0.87~1.31)	1.62 (0.99~2.64)
**Congenital malformations**			
Crude OR (95% CI)	0.52 (0.12~2.23)	0.43 (0.06~3.23)	4.40 (0.59~32.96)
Adjusted OR ^a^ (95% CI)	0.52 (0.12~2.25)	0.39 (0.05~2.92)	3.92 (0.52~29.88)
**Labor complications**			
Crude OR (95% CI)	0.79*** (0.71~.88)	0.970 (0.85~1.11)	1.72** (1.24~2.40)
Adjusted OR ^a^ (95% CI)	0.80*** (0.71~.90)	0.956 (0.84~1.09)	1.62** (1.16~2.27)

LBW, low birth weight; CI, confidence interval.

^a^ Adjusted for: maternal demographics (education, family income, urbanization level, and parity), chronic medical conditions (hyperlipidemia, thyroid dysfunction, urinary tract infections, deficiency anemia, and depression), and pregnancy history (hypertension, preeclampsia, diabetes mellitus, heart disease, and a previous cesarean section).

* *p*<0.05, ** *p*<0.01, *** *p*<0.001.

## Discussion

In this population-based study, the effects of prenatal care on adverse pregnancy outcomes in women with a history of infertility were examined. We used several measures to assess prenatal care, and considered an array of maternal demographic and health characteristics. An infertility diagnosis was found to be independently associated with more-frequent, timely, and adequate prenatal care utilization. This finding supports results of previous studies, which linked more-adequate prenatal care use with a history of infertility diagnosis and treatment [11,20]. An inability to conceive for a long time and concern about possible loss of the pregnancy might lead to higher motivation by infertile women to utilize prenatal care.

Our study results showed that women diagnosed with infertility were at increased risks of having VLBW and preterm babies, labor complications, and a CS, compared to women without infertility. These findings support the results of previous studies, which demonstrated associations of infertility with an LBW [21,22], preterm birth [21,23], obstetrical complications [21,24], and a CS [23]. Two possible mechanisms of this association were suggested, an infertility hypothesis [25,26] and an ART treatment effect [27], which still remain unclear and require further investigation. Previous studies suggested that some cesarean deliveries are not dictated by medical indications but rather by anxiety and over-concern about these desired pregnancies in women with infertility [8,9,28].

In our study, we found no statistically significant association between an infertility diagnosis and congenital malformations. A recent meta-analysis reported an increased risk of birth defects with ART pregnancies [29]. However, our finding is consistent with previous studies, which showed that the association between ART and the risk of congenital malformations was no longer significant after adjusting for age [28] and other potential confounders [30,31]. Indeed, in a recent study published in *The New England Journal of Medicine* [31], Davies at al. found that the risk of congenital malformations and other birth defects associated with an intracytoplasmic sperm injection, which is offered to infertile couples with male infertility, remained high after adjusting for parental factors. Nevertheless, it was no longer significant in in-vitro fertilization pregnancies after multivariate adjustment. This suggests that the mode of conception did not considerably bias our findings on congenital malformations among women with an infertility diagnosis.

Among pregnant women with a history of an infertility diagnosis, an inadequate number of prenatal visits was associated with a 15-fold increased risk of having a VLBW baby, and a 2-fold increased risk of an LBW or preterm baby, after adjusting for confounders. Late initiation of prenatal care was respectively associated with 2- and 1.5-fold increased risks of having VLBW and LBW babies. Our findings are consistent with previous research, which demonstrated an association between inadequate prenatal care visits and increased risks of adverse pregnancy outcomes [32-34]. The effect of inadequate prenatal care on the increased risk of adverse outcomes can be explained by detection of fewer maternal and fetal complications due to the reduced number of prenatal care visits. However, we further found that the risks of having labor complications were lower among infertile women with <6 prenatal visits, compared to infertile women with an adequate number of prenatal visits. Previous studies also suggested a possibility of an adverse self selection bias, meaning that women with the worst expected outcomes might be most likely to seek care. The effects of prenatal care might thus be underestimated [9,11]. More studies are in need to further clarify this issue. And not only the quantity, but also a clinical content of prenatal care is important in preventing adverse outcomes.

Indeed, few studies have examined the effect of the content of prenatal care on adverse pregnancy outcomes. In our study, infertile women who missed most of the laboratory tests and US examinations performed during the first 5 prenatal visits respectively had 4- and 1.6-times higher risks of having a VLBW baby and labor complications compared to infertile women with an adequate content of prenatal care. A study by Kogan et al. [19] found no difference in the risk of an LBW baby between pregnant women who received all of the recommended prenatal care procedures during the first 2 prenatal visits and those who did not receive all of them. In that study, information about the specific prenatal care procedures and health behavior advice was obtained through a patient survey. However, the authors did assume that the effect of procedures performed later during the pregnancy could have been underestimated. In our study the recommended first 5 prenatal visits with appropriate prenatal care procedures were used as a proxy measure for the content of prenatal care. In addition, we lack data on physician’s counseling services or other quality measures. Therefore, our findings should be interpreted with due caution.

Our study results suggest that an adequate number of prenatal care visits might reduce the risks of adverse birth outcomes in women with infertility. For instance, infertile women who received <6 prenatal visits had increased risks of having VLBW, LBW, and preterm babies, compared to fertile women with adequate prenatal care. However, among infertile women with an adequate number of prenatal visits, we found no significant associations with adverse birth outcomes compared to women without infertility who also received adequate prenatal care. A positive association between the number of prenatal visits and birth weight was previously reported among high-risk pregnancies [11]. The importance of adequate prenatal care should be strongly stressed, especially among women with high-risk pregnancies including an infertility history.

The major strength of this study is the comprehensive population-based nature of the dataset that included records of maternal infertility diagnoses, prenatal care, and pregnancy outcomes of 75,280 women with singleton births. A large sample size provides sufficient statistical power to detect true differences in prenatal care use and adverse pregnancy outcomes between women with and without an infertility diagnosis, who were matched by age. The study design enabled us to minimize selection, non-response, and information biases.

This study has several limitations. First, we did not differentiate between pregnancies associated with treated and untreated infertility. However, previous studies reported that there were no significant differences in perinatal outcomes between treatment-related and treatment-independent singleton births [8,21-24]. Second, specific causes of a female infertility diagnosis and its duration were not available in our claims dataset. However, results from the literature generally support the robustness of our findings. Although most previous studies were unable to assess the duration of infertility [7,8], Chung et al. indeed found no association between perinatal morbidity (preterm birth and LBW) and cause of infertility [35]. This issue was further complicated by a lack of standardized definitions for the various causes of infertility, which was often attributed to multiple causes, or the cause was classified as unknown [36]. Third, our analysis was limited to singleton live births only, and therefore multiple births and stillbirths were not represented. Fourth, our dataset only contains information on 10 free prenatal care visits, and we lack data on any additional self-paid healthcare utilization by pregnant women.

Finally, potential confounders for adverse pregnancy outcomes, such as smoking and alcohol use, were not available. However, according to previous studies, fewer women with an infertility diagnosis consumed cigarettes or alcohol during pregnancy compared to the control group [20], and adjustment did not considerably alter the odds of a premature or LBW baby [21]. Specifically, results from a large Australian population-based survey of recent mothers may be considered as an example [37]. The univariable analysis showed a significant association between smoking and an LBW (OR 1.99, 95% CI 1.3~3.0). Women who smoked throughout their pregnancy were more likely to attend fewer than 5 prenatal visits (OR 2.55, 95% CI 1.7~3.8). Although women attending fewer than 5 prenatal visits were 4.23-times (95% CI 2.6~7.0) more likely to have an LBW infant, the multivariable analysis showed that the effect of <5 visits on LBW remained significant and considerable even after adjusting for smoking and other factors (OR 3.48, 95% CI 2.1~5.8).

## Conclusions

Our study results suggest that an adequate number and content of prenatal care visits reduce the risk of adverse pregnancy outcomes in women with infertility, especially the risk of a VLBW birth. This population-based study revealed no significant association between female-caused infertility and congenital malformations. Our findings reemphasize the important role of adequate prenatal care in favorable pregnancy outcomes. It should be stressed that frequent and timely prenatal care, which provides important screening, testing, and monitoring services, has the potential to reduce the risks of adverse birth outcomes, especially among high-risk pregnancies of women with a history of infertility.

Further investigation of the quantity and quality of prenatal care is suggested in order to improve the effectiveness of prenatal care for high-risk pregnancies. Additional research is needed to differentiate the effects of prenatal visits on birth outcomes among treated and untreated women with an infertility diagnosis, and examine the association between paternal infertility and pregnancy outcomes.

## Supporting Information

Table S1 **ICD-9-CM coding utilized in this study (in the order of appearance).**
(DOC)
