# Supplementary material for: Adequate Prenatal Care Reduces the Risk of Adverse Pregnancy Outcomes in Women with History of Infertility: A Nationwide Population-Based Study
Source: PLoS One. 2013 Dec 17;8(12):e84237. doi: 10.1371/journal.pone.0084237 (PMC3866182; doi:10.1371/journal.pone.0084237)
Supplement: Table S1 — ICD-9-CM coding utilized in this study (in the order of appearance). (DOC) [file pone.0084237.s001.doc]

Table S1: ICD-9-CM coding utilized in this study (in the order of appearance)

| Disease | ICD-9-CM codes |
| --- | --- |
| Infertility, female | 628 |
| Hyperlipidemia | 272, 272.0~272.9 |
| Type 2 Diabetes | All 250 excluding type 1 diabetes |
| Thyroid dysfunctions | 242.9, 244, 246 |
| Urinary tract infections | 599.0 |
| Deficiency anemias | 280.1~281.9, 285.9 |
| Depression | 300.4, 301.12, 309.0, 309.1, 311 |
| Gestational hypertension | 642.3, 642.9 |
| Preeclampsia | 642.4 (mild preeclampsia), 642.5 (severe preeclampsia) |
| Gestational diabetes | 648.8 |
| Coronary heart disease | 410~414, 429.2, 648.6 |
